# Characteristics of Global Data on Adolescent’s Dietary Intake: A Systematic Scoping Review

**DOI:** 10.1016/j.cdnut.2023.102054

**Published:** 2023-12-01

**Authors:** Kathrin M Demmler, Ty Beal, Mona Z Ghadirian, Lynnette M Neufeld

**Affiliations:** 1 Knowledge Leadership, Global Alliance for Improved Nutrition (GAIN), Berlin, Germany; 2 Knowledge Leadership, Global Alliance for Improved Nutrition (GAIN), Washington, DC, United States; 3 School of Human Nutrition, McGill University, Montreal, QC, Canada; 4 Nutrition Division, Food and Agriculture Organization of the United Nations (FAO), Rome, Italy

**Keywords:** dietary intake, food consumption, adolescents, young adults, review

## Abstract

Data on adolescents’ dietary intake are essential to improve their diets and nutrition. However, the availability of (high-quality) data on adolescents’ dietary intake is scarce with great global differences. We conducted a systematic scoping review to investigate the availability, characteristics, and gaps in global adolescent dietary data, following the Preferred Reporting Items for Systematic Reviews and Meta Analyses—Extension for Scoping Reviews checklist and guidelines (registered under PROSPERO no. 171170 https://www.crd.york.ac.uk/PROSPERO/). We included peer-reviewed and grey literature articles (2010 onwards) on the dietary intake of male and female adolescents (10–24 y). Studies from all countries and languages and including any information related to types of food consumed, diet composition, dietary diversity, or meal patterns were considered. We excluded studies with insufficient methodological information, unclear description of population, samples sizes <25, school-based data sets containing <6 schools, and studies that focused on pregnant or unhealthy study populations. Data, including year(s) of data collection, age, gender, sample size, dietary assessment methods, number of food items/groups, study design, location, and representativeness, were extracted. A total of 52,889 titles were identified and 722 articles, describing 1,322 data sets, were retained for analysis. Nationally representative, detailed dietary data for adolescents aged 10–24 y are still lacking, particularly in sub-Saharan Africa, South Asia, and low-income countries. Data quality and representativeness remain limited, highlighting the need for data disaggregation by age, gender, locality, comprehensive dietary information, and broader geographic coverage. A notable amount of data was available through grey literature, especially in data-scarce countries. The study underscores the importance of addressing adolescent nutrition, emphasizing the urgent need for more robust, accessible, and representative data on adolescents’ dietary intake to support effective nutritional efforts.

## Introduction

Adolescence is a critical time for physical, cognitive, and emotional growth and development. Healthy adolescent diets and nutrition are of foremost importance for later adult health and future generations, and for the realization of adolescent’s potential [1]. Despite the high nutritional needs in this age group [2], worldwide dietary habits for adolescents are often characterized by the consumption of energy-dense ultraprocessed foods high in salt and refined sugar, saturated and trans-fats, breakfast skipping, and fast food with only moderate to low consumption of fruits and vegetables [[3], [4], [5]]. In many countries, adolescent underweight has persisted, whereas the mean BMI and the prevalence of adolescent overweight and obesity increased between 1975 and 2016. If current trends in adolescent overweight and obesity persist, it is likely to surpass moderate and severe underweight by 2022 [6,7].

The availability of disaggregated adolescent dietary intake data is essential across different contexts and subgroups. A better understanding of adolescent’s diets and nutrition is important to inform efforts that improve adolescent’s health and that of future generations. Improved nutrition before and during pregnancy is linked to better birth outcomes and maternal health [8,9]. However, the availability of adolescent’s dietary data is limited and knowledge gaps remain [10], harming the ability to rigorously assess adolescent’s diets and to develop evidence-based interventions.

Recent research revealed that representative full dietary details are scarce globally and disaggregated dietary data are limited for most age groups, particularly in lower- and middle-income countries (LMICs) [11,12]. According to Neufeld et al. [11], only 11% of countries, mostly high-income countries (HICs) in the Americas, Europe, or Asia, had detailed individual-level dietary intake data for adolescents at national or sub-national level. Although the researchers recognized extant dietary data for adolescents that had not been fully analyzed and synthesized in published form, the overall availability of dietary intake data for adolescents was still scarce. Keats et al. [4] found that data were particularly lacking in Africa, and in LMICs located in Europe and Central Asia (ECA). In addition, a considerable share of available data could not be disaggregated by gender [11]. Furthermore, the overall quality of available data and underlying studies was often deemed low because of limited scale and sampling methods and a lack of standard measures to assess high-quality dietary data in adolescents particularly in LMICs [4,5,13,14]. Another limitation is that data were usually of non-nationally representative nature [11,14]. A review by Newens and Walton [15] collated dietary data on sugar intakes of different population subgroups only found 14 countries—all from Europe, North America and Australasia—for which nationally representative dietary data on sugar intakes for adolescents were available.

Prompted by the evidence presented and driven by the need to address the critical gaps in the existing literature regarding an in-depth understanding of data availability, quality, and gaps, we conducted a systematic scoping review of peer-reviewed and grey literature in all languages on the diets of adolescent boys and girls 10–24 y globally. We aimed to answer the following research questions:

• What type(s) of dietary intake data are available for adolescents?
• How does availability of data on adolescents’ dietary intake differ by region, country, and income group?
• To what extent are available data disaggregated by locality (that is, urban and rural) and gender?

## Methods

We conducted a systematic scoping review to examine the nature of globally available dietary intake data for adolescents building on work that has been previously presented in Neufeld et al. [11]. A systematic scoping review was deemed the most appropriate approach because of the kind of research questions, which relate to the examination of the nature of data, existing evidence in the field of study as well as the aim to identify knowledge gaps in a systematic manner [16,17]. We reviewed peer-reviewed journal articles as well as grey literature. The development of the protocol and reporting followed the PRISMA—Extension for Scoping Reviews (PRISMA-ScR) checklist and guidelines [18] (Supplemental File A, Supplemental Table 1). This review is registered under PROSPERO no. 171170 (https://www.crd.york.ac.uk/PROSPERO/).

### Data sources and search strategy

We searched PubMed electronic database for peer-reviewed journal articles examining dietary intake for adolescents. Details of the search strategy are included in Supplemental File A. Although the search was initially carried out on 25 February, 2020, the search was updated with identical search strings on 6 December 2021 (Supplemental File A, Supplemental Table 2). In addition, a comprehensive grey literature search was conducted between June and September 2020. This included the screening of abstracts of the reports of organizations conducting research on adolescents, diets, and nutrition (e.g., United States Agency for International Development, Young Lives, UNICEF, Global Alliance for Improved Nutrition, etc.). In addition, data sets from the Global Dietary Database (GDD) and the joint Global Individual Food Consumption Data Tool (GIFT) by the FAO of the United Nations and the WHO were screened with the assistance from researchers from Tufts University and Johns Hopkins University. Furthermore, a Google and Google Scholar search on each country (including dependent territories and self-declared nations) examining dietary intake for adolescents was conducted including the first 10 results for each country (Supplementary File A, Supplemental Table 3).

After duplicates removal, all relevant titles and abstracts were screened against the inclusion/exclusion criteria (see Eligibility criteria below). In a second step, full-text papers were screened for those passing inclusion criteria using a standardized Microsoft Excel template to chart details including but not limited to year(s) of data collection, age, gender, sample size, dietary assessment methods, the number of food items/groups assessed, study design, location, and representativeness (Supplementary File A, Supplemental Table 4).

Title, abstract and full-text screening involved a total of four researchers, working independently. Corresponding authors were contacted if key data were missing. The number of excluded studies, with reason, was recorded at each stage. We analyzed the study design and data collection, the type of dietary data, the localities in which the assessments were conducted and the level of disaggregation by gender (combining biological sex and gender, for boys and girls) that was provided for the total sample of data sets, by region and country, and country income groups in which the data sets were assessed. Furthermore, we developed maps to highlight the analyzed results of data sets by region and country. The analysis and the development of maps were conducted by one researcher (KMD) using statistical software Stata 15 [19] and publicly available source maps [20].

### Eligibility criteria

We included studies that were conducted from 2010 onwards to provide a recent picture of the dietary intake data available for adolescents. We did not restrict studies by earlier years of data collection (before 2010) if data could not be disaggregated from the years of interest to allow a higher number of studies to be included. Studies from all countries (including dependent territories and self-declared nations) and in all languages, utilizing quantitative and mixed methods were included. In detail, this consisted of observational studies (e.g., cross-sectional, cohort, case-control) and randomized controlled trials (RCTs) with a control arm or baseline dietary data. Studies examining data on any sub-set of ages 10–24 y (but not restricted by upper or lower age limits and those that included either or both genders) were included. For studies that did not allow to disaggregate the age ranges of interest from a broad age range the mean age of the study population was considered. If the mean age of those studies with broad age ranges fell in the age range of interest (10–24 y) the studies were included. Instead of 10–19 y of age the age range of 10–24 y as defined by Sawyer et al. [21] was chosen to allow for an expanded and more inclusive definition of adolescence as it acknowledges earlier puberty, continued growth and a popular understanding of this life phase. Studies from which any information related to types of food consumed, dietary composition, dietary diversity, from which meal patterns could be estimated (whether quantitative, semi-quantitative, or categorical, e.g., yes/no consumption of specific food types) were included.

We excluded studies that only reported on findings in a narrative way, e.g., describing dietary styles, or studies without details on the actual foods consumed. We also excluded studies with year of data collection earlier than 2010 and with unverifiable year of data collection (even after reaching out to the corresponding author). School-based studies in <6 schools were considered as non-representative for the population and not considered. Furthermore, data sets with samples sizes <25, studies that did not provide a clear description of the population under study, and studies that focused on pregnant adolescents or adolescents with health issues or eating disorders were excluded.

## Results

### Data characterization

A total of 52,889 titles were located from the literature search (PubMed 2020 = 20,898; PubMed 2021 = 21,434; grey literature = 10,557) (Supplemental Figure 1). After removing duplicates, 27,878 unique records were identified as potentially relevant for this review. After title and abstract screen, 2,635 articles were retrieved and scanned in their entirety for eligibility, 1 additional article was identified through experts and deemed relevant, with 722 articles (*n* = 565 articles from the PubMed search in 2020 and 2021, and *n* = 157 articles from grey literature search) being retained for analysis (Supplementary File A, Supplemental Table 5). Overall, the 722 articles described data for 1,321 data sets. We define data sets as the data assessments by country within 1 article or study. Most papers (94%) contained data sets on 1 country, whereas a few (6%) contained data sets on >1 country, ranging from 2 to 65 countries per article.

### Total sample

Out of the 722 articles included, 677 (94%) were peer-reviewed and 45 (6%) were grey literature, presenting 1,223 and 98 data sets, respectively (Table 1).

**TABLE 1:** Data set characteristics of adolescent’s dietary data of the total sample, by region and by income group

Data set characteristics	Total		By region							By income group			
			EAP	ECA	LAC	MENA	NA	SA	SSA	HIC	UMIC	LMIC	LIC
	*n*	%	%	%	%	%	%	%	%	%	%	%	%
Publication type of article													
Grey literature	98	7	2	17	2	2	7	14	6	9	4	7	4
Peer-reviewed	1223	93	98	83	98	98	93	86	94	91	96	93	96
Data collection (year of initiation)^1^													
1999–2009	194	15	8	29	2	7	33	2	2	25	6	5	
2010–2014	884	67	72	60	77	74	54	61	67	60	76	72	63
2015–2020	243	18	21	10	21	18	13	37	31	15	18	22	37
Age ranges (y)^2^													
1–9	163	12	10	17	6	14	25	3	3	17	9	8	
10–12	835	63	59	75	57	60	59	56	69	64	56	67	69
13–15	1073	81	79	86	83	86	67	76	88	78	81	87	88
16–18	715	54	54	43	58	59	61	68	56	53	58	52	53
19–21	275	21	11	16	26	17	28	39	35	20	23	18	33
22–24	140	11	9	11	11	10	12	14	11	12	11	7	8
>25	101	8	7	7	8	7	9	10	10	8	8	6	6
Study design													
Cross-sectional	1252	95	96	94	97	98	88	93	97	92	97	97	96
Longitudinal	31	2	3	3	2		5		1	4	2	0	2
RCT baseline	27	2	1	2	0	2	6	5	2	3	1	2	2
Other (case-control, repeated cross-sectional)	11	1	1	2			1	2		1	1		
Sample size ranges (*n*)													
<500	191	14	9	16	15	14	16	25	19	16	15	11	16
500–<1500	253	19	15	20	20	19	17	29	23	19	18	18	31
1500-<5000	339	26	23	23	23	29	36	24	29	27	23	25	27
5000–<10,000	120	9	8	17	5	3	14	3	1	12	8	5	
10,000–<100,000	128	10	17	5	8	10	14	7	1	9	11	11	
>100,000	290	22	29	18	30	24	4	12	28	16	25	30	27
Nationally representative (yes)	750	57	73	49	67	56	38	39	55	49	60	68	57
School-based sampling (yes)	962	73	83	65	72	90	54	73	76	65	75	86	71
School-based and nationally representative (yes)	579	44	65	30	52	54	12	4	51	31	48	65	53
Type of dietary assessment^3^													
FFQ, nonquantitative	879	65	67	74	61	82	49	53	66	66	59	68	70
FFQ, semi-quantitative	224	17	19	9	15	15	19	20	18	12	22	21	12
24h dietary recall	205	15	11	11	22	2	31	25	15	16	18	10	14
Other (food diary, weight recall, photo analysis)	45	3	3	7	1	1	1	2	2	5	2	1	4
Dietary details assessed (*n*)													
≥ 7 food groups	149	11	8	11	19		13	24	12	11	15	8	14
2–6 food groups	922	70	66	83	59	77	63	64	66	72	66	70	69
1 food group	250	19	26	6	22	23	24	12	22	17	19	22	16
Available disaggregation (locality)													
Urban	107	8	6	4	12	13	9	14	7	8	11	7	6
Rural	25	2	0	1	5		1	10	3	1	3	2	2
Disaggregated (urban and rural)	92	7	9	2	10	7	1	12	16	3	8	11	18
Aggregated (urban and rural)	451	34	40	42	28	31	25	24	30	34	34	36	20
Unknown locality	646	49	45	51	45	48	63	41	45	54	43	44	53
Available disaggregation (gender)													
Boys	5	0	1	0		1	1	0	0	0	0	0	
Girls	45	3	2	1		5	2	15	15	2	1	7	20
Disaggregated (boys and girls)	389	29	28	37	24	27	32	24	23	34	27	24	27
Aggregated (boys and girls)	882	67	69	62	76	67	66	61	62	64	71	69	53

Abbreviations: EAP, East Asia & Pacific; ECA, Europe & Central Asia; HIC, high-income country; LAC, Latin America & Caribbean; LIC, low-income country; LMIC, lower-middle-income country; MENA, Middle East & North Africa; NA, North America; SA, South Asia; SSA, Sub-Saharan Africa.; UMIC, upper-middle-income country.

1 Data collected before 2010 were included if these data could not be disaggregated from the sample of interest (studies conducted from 2010 onwards).

2 Because the studies included were not restricted by upper or lower age limits of adolescents (10–24 y), data sets included children (1–9 y) and adults (>25 y) if these age groups could not be disaggregated from the sample of interest.

3 A total of 32 data sets used 2 dietary assessment methods; hence, the total for dietary assessments methods was 1,353.

### Study design and data collection

Most of the dietary data were collected in cross-sectional surveys (95%) with 59% having sample sizes of ≤5000. However, 290 data sets (22%) had surveys with sample sizes ≥100,000. Over half of all data sets (57%) were from nationally representative surveys, collected through cross-sectional surveys (99%), and 73% were collected through school-based assessments, 44% were from school-based assessments, including nationally representative samples of school-going children.

Most data (67%) were collected between 2010 and 2014, including adolescents of the age ranges between 10–12 y (63%), 13–15 y (81%) and 16–18 y (54%). Less often, older adolescents of age ranges between 19–21 y (21%) and 22–24 y (11%) were included. Children aged <10 and >24 y were included in 12% and 8% of the data sets, respectively, but without ability to disaggregate from the broader age range given as inclusion criteria.

### Type of dietary data

Nonquantitative food frequency questionnaires (FFQs), that is, assessments that aim to capture the usual dietary consumption of pre-defined food items or food groups over a designated time period, typically capturing the frequency but not portion sizes [22], were the most common dietary assessment method (65%). Other methods used included semi-quantitative FFQs (17%), i.e., FFQs that in addition to capture usual or specific portion sizes, 24-h dietary recalls (15%), and other methods including food diaries, weighed dietary recall or photo analysis methods (3%). Seventy percent of the data sets captured dietary details on 2–6 food items/groups, 19% captured dietary details on only 1 food item/group, and 11% on ≥7 food items/groups. Those data sets capturing only 1 food item/group were mostly on particular foods of interest, e.g., fast food or sugar-sweetened beverage consumption. Most of the data sets capturing only 1 or between 2 and 6 food items/groups were assessed using nonquantitative FFQs (79% and 71%, respectively) whereas those capturing dietary details on ≥7 food items/groups came mostly through 24-h dietary recalls (78%). Out of the dietary data sets with ≥7 food items/groups less than half (49%) were nationally representative. For data sets with between 2 and 6 or only 1 food item(s)/group(s) the shares of nationally representative data were 54% and 70%, respectively.

### Locality and gender

Almost half of the data sets had unknown locality details (49%) or displayed aggregated data on urban and rural localities (34%). Only 7% of data sets allowed for data disaggregation by locality, i.e., displaying data on both urban and rural areas, 8% were from urban localities only and 2% from rural localities only. Disaggregated dietary data by gender, i.e., including data on both girls and boys, was available for 29% of data sets. Overall, a small share of data sets displayed dietary details on either girls (3%) or boys (0.4%) only. Most of the data sets (67%) displayed aggregated dietary data, i.e., not differentiating between girls or boys.

### By region and country

There were 1,321 data sets that provided data for 153 countries (61% of the list of 249 countries and territories worldwide [23]) and 7 world regions (Supplementary File A, Supplemental Table 6), as defined by World Bank [24]. Of the data sets only 8% and 4% were from sub-Saharan Africa (SSA) and South Asia (SA), respectively.

Although the share of data sets from peer-reviewed articles was dominant in all regions, Table 1 depicts that ECA had a substantial share of data sets from grey literature (17%), almost all of which were identified through the screening of GDD (98%).

**FIGURE 1:**
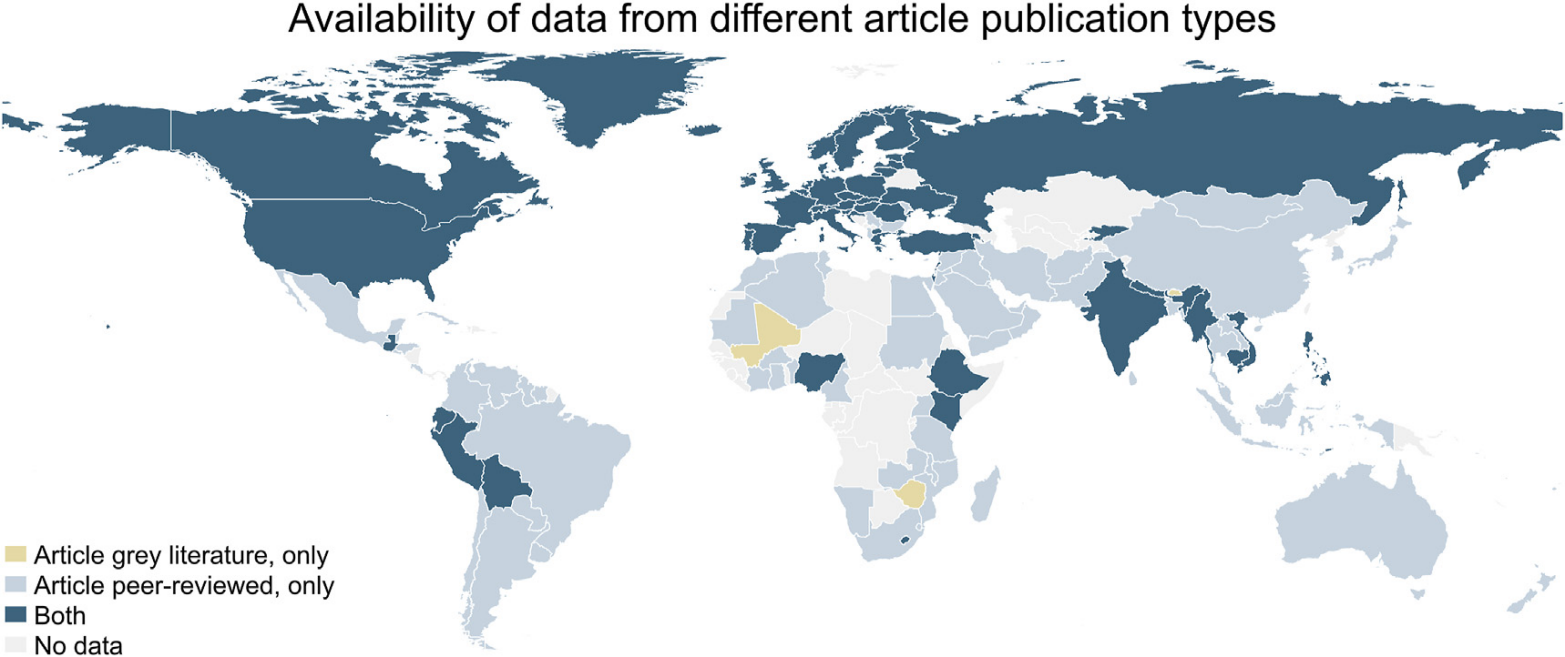
Availability of different article publication types of adolescent’s (age 10–24 y) dietary data per country, 1999–2020.

Figure 1 shows that for 4 countries (Bhutan, Mali, Malta, and Zimbabwe) the only available adolescent’s dietary data was from grey literature. Almost two-third of countries (95 countries; 62%) had only data sets from peer-reviewed articles; 54 countries (35%) had data from both publication types available.

### Study design and data collection

**FIGURE 2:**
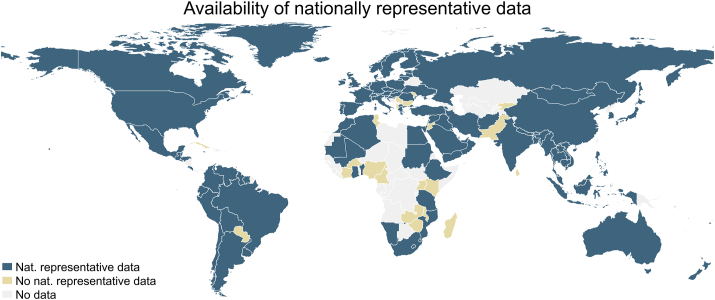
Availability of nationally representative adolescent’s (age 10–24 y) dietary data per country, 1999–2020.

Although most studies were of cross-sectional design, a small share of surveys in NA were RCT baselines (6%) or of longitudinal design (5%). The share of data sets with sample sizes of ≤5000 was particularly high in SA (78%) and SSA (70%). About one-third of data sets in Latin America and Caribbean (LAC), EAP, and SSA included samples of ≥100,000. The share of nationally representative data was comparatively low for SA (39%) and North America (NA) (38%) and rather high for East Asia and Pacific (EAP). In addition, Figure 2 shows that globally 14% of all countries (*n* = 22) did not have any nationally representative adolescent’s dietary intake data available, most of them were countries in SSA (*n* = 10) and ECA (*n* = 6).

**FIGURE 3:**
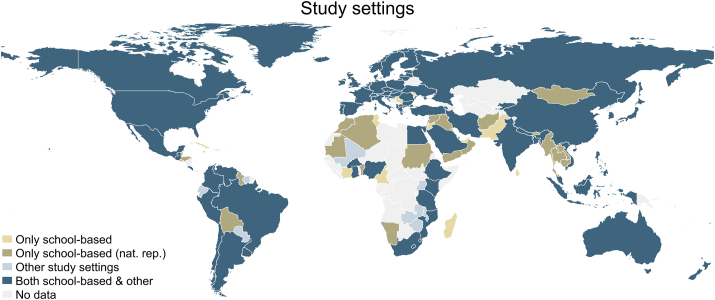
Study settings including school-based assessments and other of available adolescent’s (aged 10–24 y) dietary data per country, 1999–2020.

The share of data sets assessed at school was particularly high in Middle East and North Africa (MENA) (90%) and EAP (83%). Both regions also had the highest shares of data that included nationally representative school-based samples 54% and 65%, respectively, whereas NA and SA had lowest shares of 12% and 4%, respectively. Globally, for 43% of all countries (*n* = 66) the available adolescent’s dietary data was solely based on school-based sampling (*n* = 18) and school-based sampling using a nationally representative sample (*n* = 48) (Figure 3).

The year of data collection initiation was mostly between 2010 and 2014, however NA and ECA had sizable shares (33% and 29%, respectively) of data collected earlier, that is, between 1999 and 2010. SA and SSA had the highest shares of data collected in more recent years, that is, between 2015 and 2021 (37% and 31%, respectively). The age ranges of included adolescents were similar for all regions; however, a comparatively high share of adolescents between 19–21 y of age was included in data sets in SA (39%) and SSA (35%), whereas in NA ∼25% of data sets also included aggregated data on children aged <10 y.

### Type of dietary data

**FIGURE 4:**
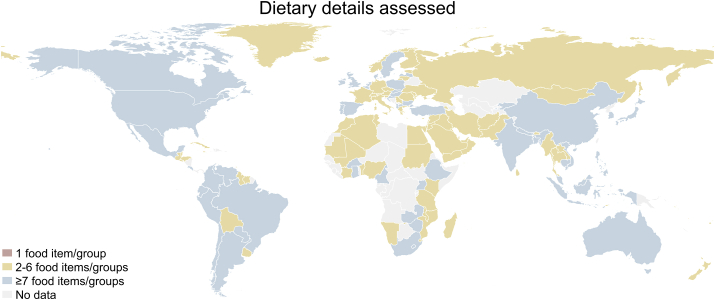
Available dietary details assessed on adolescent’s (aged 10–24 y) per country, 1999–2020.

Nonquantitative FFQs were the most prominent dietary data assessment method in all regions. In NA and SA about one-third of data sets were further assessed using 24-h dietary recalls. The share of data sets capturing dietary details of ≥7 food items/groups was highest in SA (24%) and lowest in EAP (8%) and MENA, where none of the data sets captured >2–6 food items/groups. On country level, available data for 3 countries (American Samoa, Guam, and Palau) included only dietary details on 1 food item/group; 98 countries had dietary details on 2–6 food items/groups; 52 countries had dietary data with details on ≥7 food items/groups available (Figure 4).

### Locality and gender

**FIGURE 5:**
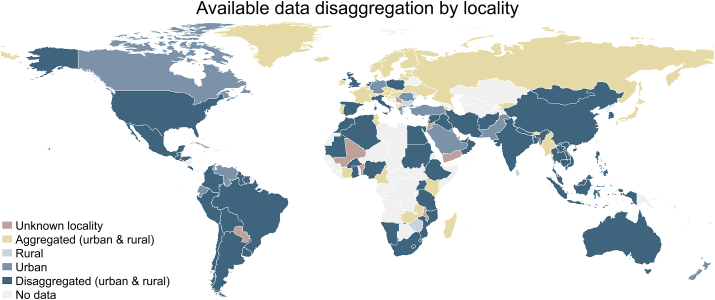
Available adolescent’s (aged 10–24 y) dietary data disaggregation by locality (urban and rural) per country, 1999–2020.

For all regions, particularly NA and ECA, the share of adolescent’s dietary data sets disaggregated by locality (urban and rural) was low. However, considering all available data at country level, 47% of countries (*n* = 72) had at least some adolescent’s dietary data disaggregated by locality; 11 countries (7%) had only data with unknown locality details; 54 countries (35%) had either unknown or aggregated locality details available (Figure 5). In addition to unknown and aggregated locality details, 16 countries (10%) had data on either rural (*n* = 5) or urban (*n* = 11) areas available.

**FIGURE 6:**
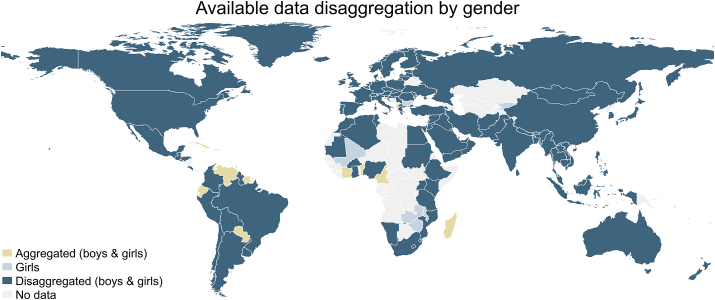
Available adolescent’s (aged 10–24 y) dietary data disaggregation by gender (combining biological sex and gender, for boys and girls) per country, 1999–2020.

Although the shares of data sets including disaggregated data by gender ranged between 23%–37% for all regions, 80% of the countries (*n* = 123) had some disaggregated adolescent’s dietary data available; 23 countries (15%) had only aggregated data; 7 countries (5%) had aggregated data and/or data on girls available (Figure 6).

### By income group

Almost half of all data sets retrieved (48%) stem from HICs, followed by upper-middle-income countries (UMIC) (24%) and LMIC (24%). Only 4% of all data sets capture findings from low-income countries (LICs), as defined by World Bank [25]. As depicted in Table 1, the share of peer-reviewed data sets ranged between 91%–96% for all income groups.

### Study design and data collection

Above all income groups the major study design was cross-sectional. However, the share of data sets with sample sizes of ≤5000 was particularly high for LICs (73%) and the share of large samples with ≥100,000 was comparatively high in LMICs (30%) and UMICs (25%). Interestingly, the share of data sets being nationally representative and data sets assessed in school settings were both lowest for HICs (49% and 65%) and highest in LMICs (68% and 86%). Also, the share of school-based assessments including nationally representative samples of adolescents was higher for LMICs (44%) compared with HICs (31%).

UMICs, LMICs and LICs had low shares of data collected between 1999 and 2010 (6%, 5% and 0%, respectively) and LICs a particularly high share of data being collected in more recent years, that is, between 2015 and 2021 (37%). The assessed age ranges did not show major differences by income groups. However, LICs had a comparatively high share of assessed dietary data for adolescents aged between 19 and 21 y (33%).

### Type of dietary data

Nonquantitative FFQs were the most-used dietary assessment method in all income groups. The share of other methods used, including semi-qualitative FFQs and 24h dietary recalls, was highest in the data sets in UMICs (40%). LMICs had the lowest share of data sets with dietary details of ≥7 food items/groups (8%) and the highest share of data sets with details on only 1 food item/groups (22%).

### Locality and gender

The share of data sets disaggregated by locality (urban and rural) was particularly low for HICs (3%) and highest for LICs (18%). The share of data sets disaggregated by gender (combining biological sex and gender for boys and girls) was highest for HICs (34%).

## Discussion

The findings of this systematic scoping review underline that data on adolescent’s dietary intake are still scarce. For over one-third of all countries worldwide no adolescent’s (aged 10–24 y) dietary data were found through our literature search, capturing peer-reviewed and grey literature published after 2010. Most of the countries where no data were found were in SSA.

This scoping review found a substantial number of countries that did not have any nationally representative data. Those countries were mostly in SSA and ECA. Furthermore, particularly for those data sets that contained ≥7 food items/groups less than half of them were nationally representative, indicating that even if nationally representative data for adolescents were available the dietary details captured were often limited. Focusing on nationally representative dietary data from public and private surveys for the GDD 2017 update, Miller et al. [26] found that for all regions except SA, most surveys were nationally representative. Although the GDD project expanded the focus to include adolescents besides infants, children, and adults, only 50% and 61% of the identified data represented adolescents between 11 and 14 y and 15 and 19 y of age, respectively. Although the authors highlight the possibility that these shares might be higher given that data on children and adolescents (0–19 y) were not collected in 2010, the shares underline the limited availability of nationally representative dietary data for adolescents. Give the scarcity of nationally representative studies, Keats et al. [4] did not limit their systematic review of dietary intake and practices of adolescent girls in LMICs to such type of data. All studies underline the crucial need for nationally representative dietary data for public health and policy development, serving as a foundation for informed decision-making and as basis for the monitoring and evaluation of the Sustainable Development Goals [4,26,27].

Some of the available data on adolescent’s dietary intake are published in the grey literature, particularly for SSA, and for the older adolescent age subgroups. Grey literature publications may be less likely to be peer-reviewed. They may be more accessible (e.g., not behind journal paywalls), but more difficult to find (e.g., not indexed in major bibliographic resources). However, they were the only data available for several countries, and are an important source of data and evidence for nontechnical readers. The importance of grey literature and the need to ensure quality, visibility, accessibility and preservation has been identified [28]. Hence, methods are required to promote open access, automated processes and online data cataloging, to enhance visibility and accessibility of high-quality (grey) literature [29].

In addition to the data gaps, our review identified important limitations of existing data for almost all countries worldwide. Data for older adolescents (19–24 y of age) and data disaggregated by gender (i.e., boys and girls) and locality (that is, urban and rural) were scarce, particularly for EAP, and ECA, and LMICs. Many countries in EAP, MENA and LAC, and in UMICs and HICs, had only adolescent’s dietary data collected through school settings. Much of the school-based data were collected as part of the Global School-Based Student Health Survey (GSHS) developed and implemented by WHO [30]. The initiative was designed to gain internationally comparable data and support countries to assess key areas of adolescent’s behavioral risk factors and protective factors with a low-cost survey. Although the initiative added tremendously to the available representative dietary data for adolescents there are several limitations. The nonquantitative FFQs used under the GSHS capture the frequency of consumption during the last 7 days, but only on a very limited number of food groups (fruits, vegetables, carbonated soft drinks, sugar-sweetened drinks), and for a limited age range of adolescents (13–17 y old) [30]. Given the nature of assessment within schools, the surveys may exclude a large proportion of the adolescent population. A study in HICs identified significant positive effects on school dropouts related to multiple child, parent, family, peer, and school factors, e.g., low IQ and learning difficulties [31]. Studies from LMICs settings identified female adolescents to be more likely to leave school at younger ages because of household responsibilities, early pregnancy or marriage. Furthermore, Johnson et al. [32] argued that school dropouts may be related to poverty and food insecurity whereas school attendance has been associated with fewer health risk behaviors and better health outcomes. Going forward it will be important to develop new approaches to assess dietary intake of all adolescents—whether in school or not—to develop tailored programs and initiatives to promote healthier diets. Unfortunately, our review highlights critical gaps for most countries to meet this objective.

In all regions, particularly in LMICs and LICs, FFQs were the predominant method used to assess dietary data among adolescents. FFQs are cost effective, less time-consuming, and less burdensome, offering an advantage in capturing adolescents' typical diets with extended recall periods, unlike the typically more resource-intensive 24-h dietary recalls or weighed food records [33]. However, FQQs typically capture the frequency of food intake but often lack measurements of portion sizes, raising concerns about their accuracy compared with other dietary assessment methods like 24h dietary recalls or weighed food records. The validity of FFQs varies: some studies have shown good reliability and (relative) validity of FFQs targeted to adolescents [[34], [35], [36]], whereas others have reported weaker relative validity, particularly in estimating energy intake compared with 24-h dietary recalls, food records and weighed food records [37]. The accuracy of FFQs is influenced by factors, such as the representation of local and culturally specific foods, the number of food items included, the interval between assessments, and whether FFQs are self-assessed or interviewer-assessed [35,38,39]. It is important to note that all dietary intake measures inherently involve some level of uncertainty [40]. Therefore, the development and use of FFQs, including the selection of food items and quantities, should ideally undergo validation for each new assessment, local context, and target population [41]. Unfortunately, this validation step is often overlooked [42] and the ability of FFQs to accurately quantify nutrient intake and provide meaningful insights into nutrient adequacy can be limited [41].

Our review also found a limited number of dietary details assessed. A substantial share of countries had adolescent’s dietary details available on only <7 food items/groups, and only simple response options with no ability to quantify intake of these few options. In the absence of a validated score to assess dietary diversity for adolescents we based our assumption on the Minimum Dietary Diversity (MDD) score for children aged 6–23 mo [43,44], which includes 7 food groups plus breastmilk. However, we are aware that other dietary indicators like the Minimum Dietary Diversity for Women (MDD-W), validated for women aged 15–49 y old, require the assessment of 10 food groups [45]. Although the MDD for children and the MDD-W have both been validated for the respective target populations and shown to be positively associated with micronutrient adequacy [[46], [47], [48]], the thresholds chosen for this review did not differentiate the actual food groups consumed, nor has it been validated and can hence not claim nutrient adequacy. However, the threshold of a 7 food group count was chosen presuming that it would be the minimum needed to provide any meaningful characterization of adolescent’s dietary intake. Our review showed that for most regions and countries (except in North America) it is not feasible to do so with the existing data.

Going forward it will be of foremost importance particularly for countries in SSA and SA and LICs, but also for other countries and income groups to increase the availability of high-quality adolescent’s dietary data. Insightful dietary details should be nationally representative, using valid survey design and data collection methods and assessing a sufficient number of dietary details to allow for the calculation of diet quality indicators, including nutrient adequacy. Furthermore, dietary data need to allow for representative disaggregation by age, locality, and gender.

Various initiatives aim to address these challenges, particularly supporting LMICs through the development and standardization of relevant indicators and tools, as well as data synthesis for evidence-based policymaking [40]. The International Dietary Data Expansion (INDDEX) project by Tufts University seeks to support LMICs to overcome high costs, inaccessibility, low quality, and under-use of household- and individual-level dietary data [49]. For example, the project developed the INDDEX24 Dietary Assessment Platform to support the collecting and assessing of actionable dietary data. By 2022, this platform was transferred to Intake—Center for Dietary Assessment at FHI Solutions (https://www.intake.org/). Together with university partners, Intake also supported the development of the Global Diet Quality Score (GDQS), a metric to measure overall diet quality across populations. The GDQS measures include categorical information about the quantities (g/day) of 25 food groups: 16 healthy food groups, 7 unhealthy food groups, and 2 food groups (red meat, high-fat dairy) consumed and offers an app for efficient and large-scale data collection [50]. The score has been validated for both nutrient adequacy and the risk factors associated with non-communicable diseases in nonpregnant, nonlactating women between 15–49 y from low-, middle-, and high-income settings [51]. The GIFT (https://www.fao.org/gift-individual-food-consumption/en), managed by FAO and WHO, offers representative individual quantitative food consumption data at the national or sub-national level. The repository can be searched by country, year, geographic coverage, residence (urban compared with rural), sex, age, and food groups. As of 2023, 45 data sets were identified including any subgroups of adolescents between 10 and 24 y; however, only 23 of them included data for boys [52]. Another recent effort is the Global Diet Quality Project (www.dietquality.org), which has collected dietary data on individuals ≥15 y in 56 countries between 2021 and 2022 and will collect data in over 30 additional countries in 2023 [53]. The data include yes/no responses to 29 food groups which enable the calculation of a suite of indicators on diet quality. These data can be disaggregated by older adolescents 15–24 y and include both boys and girls. Although the approach is an important step in the right direction it will not fully address all gaps highlighted as the data exclude the younger adolescents 10–14 y and do not provide quantitative dietary intake data in terms of quantities of grams and calories consumed. The GDD (https://www.globaldietarydatabase.org/) models the mean intake of 54 dietary factors, including foods, (e.g., fruit in g per day), beverages, macro- and micronutrients, using public and private data sources. These data are estimated for 185 countries worldwide and can be disaggregated by year, age, sex, residence (urban compared with rural), and education, allowing for the disaggregation of age groups from 10–14, 15–19 and 20–24 y [54].

### Strengths and limitations

To our knowledge, this is the first scoping review that aims to characterize dietary data for adolescent boys and girls (10–24 y) including data sets from peer-reviewed journal articles and grey literature on all countries and all languages worldwide. A systematic approach was undertaken following the PRISMA-ScR reporting guidelines. However, we acknowledge several limitations. Given the large number of results and for feasibility reasons we limited the search of peer-reviewed journal articles to 1 database (PubMed). However, we believe that the extensive search of several grey literature databases and the large number of literature from these sources provided a particular strength for our approach, given that dietary data are often unpublished. Because of the high number of identified articles we focused on food groups (e.g., vegetables) and some selected food items if they were considered relevant for a food group, e.g., milk. However, we did not include studies on individual foods (e.g., broccoli) and those that only provided insights on energy consumption or nutrients without displaying the food items/groups consumed. Furthermore, our analysis did not include the assessment of food items/groups consumed by adolescents as it has not been the scope of this review. Given the need for robust insights into dietary details of food items/groups consumed and the assessment of nutrient adequacy, future research in this area would be beneficial.

## Conclusion

This systematic scoping review highlighted that nationally representative detailed adolescent’s dietary data are still scarce, particularly in SSA and SA and all LICs. Worldwide, limited data quality was found in adolescent’s dietary data with the need for representative disaggregation by age groups, gender, and locality, capturing of dietary details, and geographic representation. Interestingly, this study identified a small but meaningful share of data sets through grey literature. Although these data are unreviewed, grey literature data can add valuable information, particularly in countries where data are scarce.

There is an increasing salience to address adolescent nutrition in recent years. However, more needs to be done as effective efforts may be constrained unless the gaps in representative, high-quality, accessible data on what adolescents eat are addressed.

## Author contributions

The authors’ responsibilities were as follows – KMD, TB, LMN: designed research; KMD, MZG: conducted research; KMD: analyzed data and wrote the paper with essential contributions from TB, MZG and LMN; KMD: had primary responsibility for final content; and all authors: read and approved the final manuscript.

## Conflict of interest

The authors report no conflicts of interest.

## Funding

This work received funding support from Fondation Botnar. The organization did not play any role in writing the manuscript or the decision to submit for publication.

## Data availability

Data described in the manuscript, code book, and analytic code will be made available upon request pending non-commercial use.
